# Novel Antioxidant Caffeate Esters of Black Locust (*Robinia pseudoacacia* L.) Stem Bark

**DOI:** 10.3390/plants15152347

**Published:** 2026-07-30

**Authors:** Ágnes M. Móricz, Anna Cselőtey, Márton Baglyas

**Affiliations:** 1 Plant Protection Institute, HUN-REN Centre for Agricultural Research, Fehérvári út 132–144, H-1116 Budapest, Hungary; cselotey.anna@atk.hun-ren.hu; 2 Doctoral School, Semmelweis University, Üllői út 26, H-1085 Budapest, Hungary

**Keywords:** black locust (*Robinia pseudoacacia*), antioxidant activity, effect-directed analysis, DPPH–HPLC–DAD–ESI-MS, HPLC–HR-ESI-MS/MS, triterpene caffeates, fatty alcohol caffeates, fatty alcohol ferulates

## Abstract

Antioxidant profiling of black locust (*Robinia pseudoacacia* L.) stem bark was performed by 2,2-diphenyl-1-picrylhydrazyl (DPPH) assay off-line coupled to high-performance liquid chromatography–diode array detection–electrospray ionization mass spectrometry (DPPH–HPLC–DAD–ESI–QMS). The ethyl acetate, methanol, and *n*-hexane extracts exhibited similar chemical profiles for the antioxidant amphipathic phenolic compounds; however, the ethyl acetate extract was the richest in them, and *n*-hexane yielded several compounds in higher ratios. A total of 21 compounds displayed antioxidant activity and were further characterized by HPLC–high-resolution tandem MS (HRMS/MS). Nine compounds were identified as previously described antioxidants from the black locust stem bark, including 3-*O*-caffeoyl oleanolic acid (**1**), and the oleyl (**4**), octadecyl (**6**), gadoleyl (**7**), eicosanyl (**9**), (*Z*)-9-docosenyl (**10**), docosyl (**12**), tetracosyl (**15**), and hexacosyl (**17**) caffeates. Further ten caffeate and two ferulate esters were tentatively identified as 3-*O*-caffeoyl oleanolic, ursolic, or betulinic aldehyde (**2**), and hexadecyl (**3**), heptadecyl (**5**), hydroxy-hexacosyl (**8**), henicosyl (**11**), tetracosenyl (**13**), tricosyl (**14**), hexacosenyl (**16**), octacosenyl (**18**), and octacosyl (**20**) caffeates, and hexacosyl (**19**) and octacosyl (**21**) ferulates that were reported for the first time in *R. pseudoacacia*. Furthermore, this is the first report about the antioxidant activity of the previously undescribed compounds **8**, **13**, **16**, and **18**, as well as known compounds **11** and **14**.

## 1. Introduction

**Figure 1:**
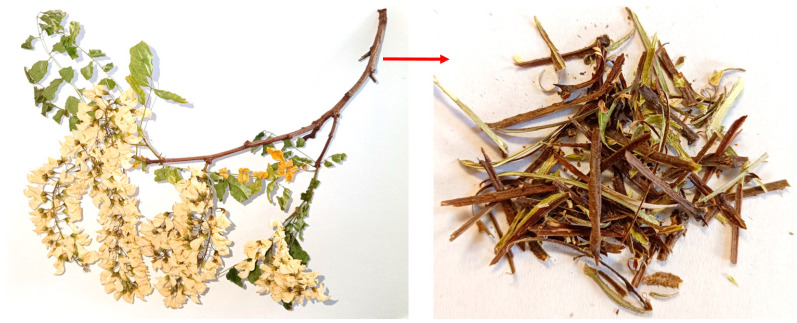
Black locust (*Robinia pseudoacacia*) blooming branch (**left**) and stem bark (**right**).

The black locust (*Robinia pseudoacacia* L.) is a deciduous tree belonging to the legume family (Fabaceae) and is native to North America. It was planted all over the world as an ornamental tree or for fuelwood or to stabilize the soil [1,2]. The plant spreads rapidly through root suckers and numerous seeds and can displace native plants, reducing biodiversity; therefore, it is considered an invasive species in many regions of the world [3]. The tree can grow to a height of up to 25 m and is characterized by pinnate leaves with oval leaflets and white, strongly fragrant, nectar-rich flowers arranged in hanging clusters between May and June, which are highly valued in beekeeping [2]. Its bark is grey-brown and deeply furrowed on the trunk, with thorns on young branches as a characteristic feature (Figure 1). The flowers, used in traditional herbal medicine to treat mild colds and gastrointestinal complaints, are rich in antioxidant phenolics, including flavonols, phenolic acids, and anthocyanins [4]. The bark is also a valuable source of bioactive phytochemicals, containing antioxidant flavonoids (e.g., catechin and epicatechin), phenolic acids (e.g., caffeic acid and ferulic acid), and tannins [5]. However, particularly the bark, but also the seeds and leaves, contain cytotoxic proteins (lectins) such as robin and phasin that can cause nausea, abdominal pain, and circulatory problems if ingested [6,7,8].

The biological role of enzymatic (e.g., superoxide dismutase, catalase, and glutathione peroxidase) and non-enzymatic (e.g., ascorbic acid, tocopherols, carotenoids, and polyphenols) antioxidants lies in the protection of cellular structures, and the prevention of oxidation-induced tissue damage and chronic diseases such as atherosclerosis, diabetes mellitus, neurodegenerative disorders, and cancer [9,10]. Antioxidants reduce oxidative stress by donating electrons and/or hydrogen atoms to reactive oxygen species (ROS), thereby neutralizing ROS and interrupting undesired oxidative chain reactions [11]. Plants can be protected from environmental influence by phenolics such as phenolic acids, flavonoids, stilbenes, and lignans, which act as antioxidants by neutralizing free radicals, quenching singlet oxygen, and chelating metals [12,13].

The combination of antioxidant assays with liquid chromatography, such as high-performance liquid chromatography (HPLC) or high-performance thin-layer chromatography (HPTLC), offers numerous advantages for the screening of individual antioxidant compounds in complex matrices, including foods, plants, pharmaceuticals, and natural products. By integrating compound separation, detection, and antioxidant activity assessment into a single analytical workflow, these methods enable the efficient identification of bioactive constituents, which is particularly valuable in the search for novel natural antioxidants [14]. Even minor constituents present in the sample can be detected when they exhibit strong antioxidant activity. To exploit this approach, HPTLC has been coupled with antioxidant assays such as 2,2-diphenyl-1-picrylhydrazyl (DPPH) and 2,2′-azino-bis(3-ethylbenzothiazoline-6-sulfonic acid (ABTS) [15]. Similarly, HPLC was combined with antioxidant assays both off-line pre-column [16,17,18,19] and on-line post-column [20,21] DPPH, ABTS, Ferric Reducing Antioxidant Power (FRAP), and Cupric Reducing Antioxidant Capacity (CUPRAC) assays. The off-line combination of HPLC and pre-column antioxidant testing relies on a conventional HPLC technique and is therefore technically simpler than the on-line HPLC–antioxidant assays, which require more complex equipment, including an additional reagent-delivery pump, a reaction coil, and a UV–Vis detector. The analytical workflow can be extended by characterizing the detected antioxidants with further chromatographic hyphenations such as HPLC/HPTLC–UV–mass spectrometry (MS) [17,18,22].

Recently, in our laboratory, HPTLC–DPPH screening revealed nine antioxidant caffeate esters in the methanol extract of *R. pseudoacacia* stem bark. The detected compounds were characterized by HPTLC–HRMS and, after isolation, were unambiguously identified by nuclear magnetic resonance (NMR) spectroscopy as 3-*O*-caffeoyl oleanolic acid and eight fatty alcohol caffeates [22]. Three of them (oleyl, gadoleyl, and (*Z*)-9-docosenyl caffeates) were identified as new natural products, and further five caffeates (octadecyl, eicosanyl, docosyl, tetracosyl, and hexacosanyl caffeates) were described for the first time in the black locust.

Building on these findings, this study aimed to further explore antioxidant constituents in the *R. pseudoacacia* bark extracts (*n*-hexane, ethyl acetate, and methanol) using an off-line DPPH–HPLC–UV–ESI-MS and HPLC–ESI-HRMS/MS.

## 2. Results and Discussion

### 2.1. Extraction and Detection of Antioxidant Black Locust Compounds

*Robinia pseudoacacia* stem bark extracts prepared with solvents of different polarity (methanol, ethyl acetate, and *n*-hexane) were screened for amphipathic phenolics with both distinct polar and nonpolar regions by RP-HPLC–DAD-ESI^−^-QMS. Such compounds are often underrepresented in conventional polar extraction-based phytochemical studies. During RP-HPLC method development, it was observed that conventional organic modifiers revealed significant limitations: acetonitrile proved to be an insufficiently strong eluent for these highly lipophilic compounds, the elution strength of methanol enabled their separation under extreme conditions, while isopropanol resulted in unsatisfactory peak shapes and poor chromatographic resolution. Substituting these traditional HPLC eluents with ethanol not only provided the necessary elution strength and resolved the chromatographic issues—yielding sharp, symmetrical peaks—but also offered a greener alternative to highly toxic organic solvents. This modification significantly reduced the environmental footprint and occupational hazards of the analytical process while simultaneously maximizing the separation efficiency of analytes with low-polarity features.

**Figure 2:**
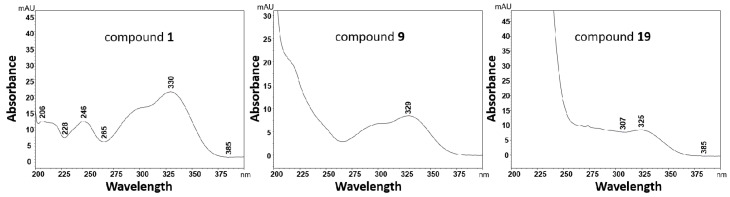
HPLC–UV spectra of *Robinia pseudoacacia* stem bark compounds **1** (*t*_R_ = 3.5 min), **9** (*t*_R_ = 15.9 min), and **19** (*t*_R_ = 33.5 min).

**Figure 3:**
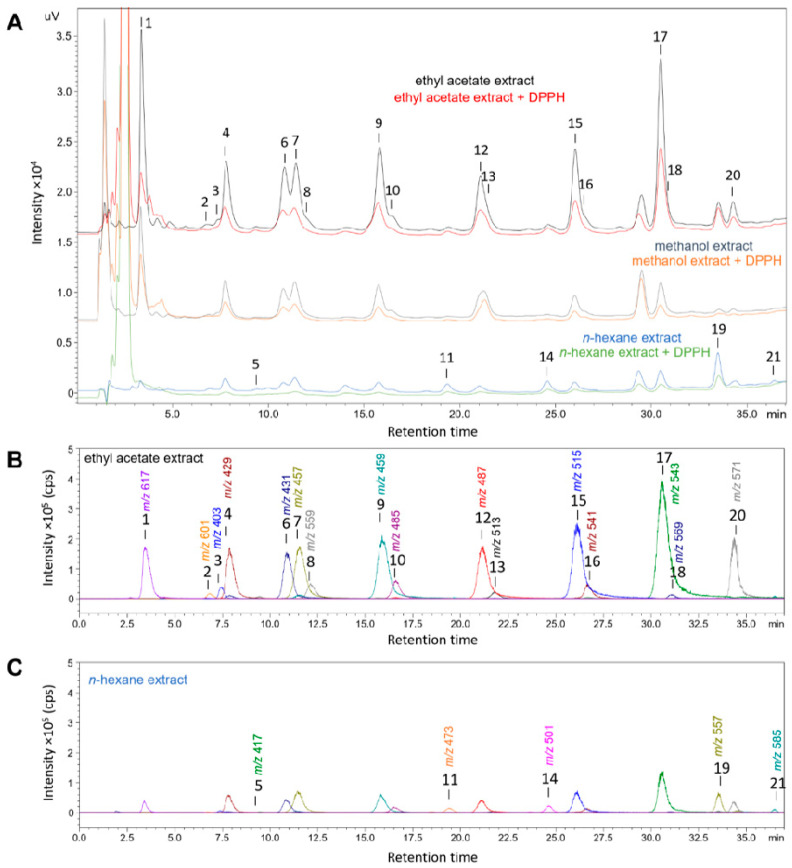
HPLC–UV chromatograms of *Robinia pseudoacacia* stem bark ethyl acetate (ten-fold diluted), methanol (ten-fold diluted), and *n*-hexane extracts before and after DPPH treatment recorded at 325 nm (**A**), and the HPLC–ESI^−^-QMS extracted ion chromatograms (EICs) of antioxidant compounds (**1**–**21**) of ethyl acetate (**B**) and *n*-hexane (**C**) extracts with their corresponding *m*/*z* values ([M−H]^−^).

**Figure 4:**
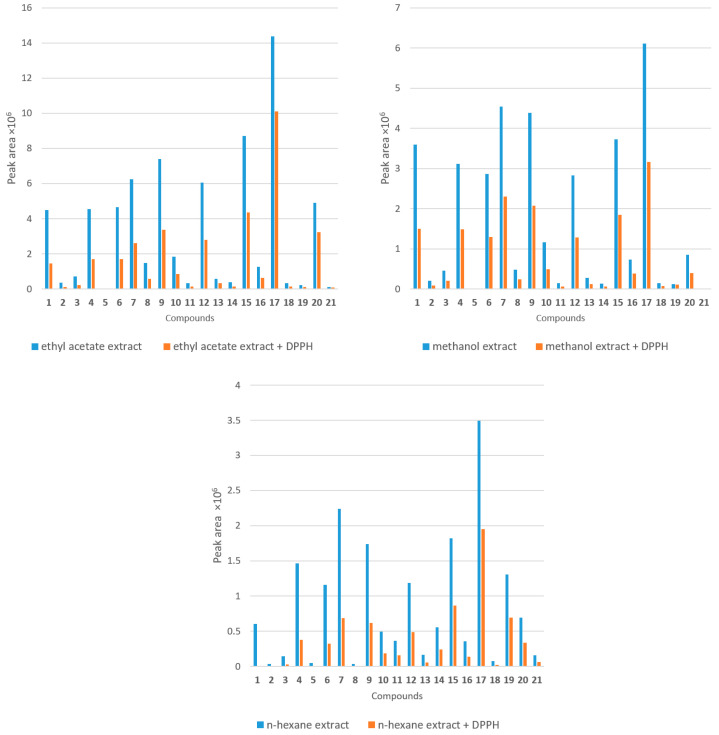
Peak areas of the antioxidant compounds (**1**–**21**) in *Robinia pseudoacacia* stem bark ethyl acetate (ten-fold diluted), methanol (ten-fold diluted), and *n*-hexane extracts (two-fold diluted) before and after DPPH treatment, obtained by the integration of the HPLC–ESI^−^-QMS extracted ion chromatograms (EICs).

The phenolic compounds exhibited characteristic UV spectra with a prominent local absorption maximum between 325 and 330 nm, as exemplarily shown in Figure 2 for three selected compounds. This spectroscopic feature can be attributed to hydroxycinnamic acids (e.g., caffeic acid, ferulic acid, and *p*-coumaric acid) and their derivatives that consist of a single aromatic ring conjugated with a side chain [23]. The separation of the compounds with low-polarity character detected at 325 nm was achieved using an ethanol gradient under acidic conditions (containing 0.1% HCOOH). The antioxidant effect of the separated compounds was assessed using an off-line DPPH–HPLC assay. Based on DPPH–HPLC–DAD–ESI^−^-QMS analysis, 21 compounds exhibited antioxidant activity, as evidenced by a reduction in peak intensity in the UV chromatogram upon treatment with the stable free radical DPPH (Figure 3A). Comparison of extraction solvents revealed that the chemical profiles of the ethyl acetate and methanol extracts were highly similar; however, ethyl acetate was more efficient at extracting antioxidant constituents than methanol (Figure 3A). In contrast, *n*-hexane showed the lowest extraction efficiency, although it yielded compounds **5**, **11**, **14**, **19**, and **21** (Table 1) in higher ratios than ethyl acetate and methanol (Figure 3A). The detection of these compounds across all extraction systems suggests that they have amphiphilic character, and the marked differences in extraction efficiency among solvents can be attributed to the polarity-dependent partitioning of the analytes. All 21 antioxidant compounds were detectable by ESI-MS, giving intense mass signals for their deprotonated molecules [M−H]^−^ in the negative ionization mode (Figure 3B,C). The peak areas of antioxidant components of ethyl acetate (ten-fold diluted), methanol (ten-fold diluted), and *n*-hexane (two-fold diluted) extracts, obtained by integrating the corresponding extracted ion chromatograms (EICs), clearly indicate the order of the solvent extraction efficiency (Figure 4). Furthermore, the peak areas became consistently lower in DPPH-treated samples compared to controls (Figure 4), confirming their antioxidant activity.

### 2.2. Identification of the Antioxidant Compounds

**Figure 5:**
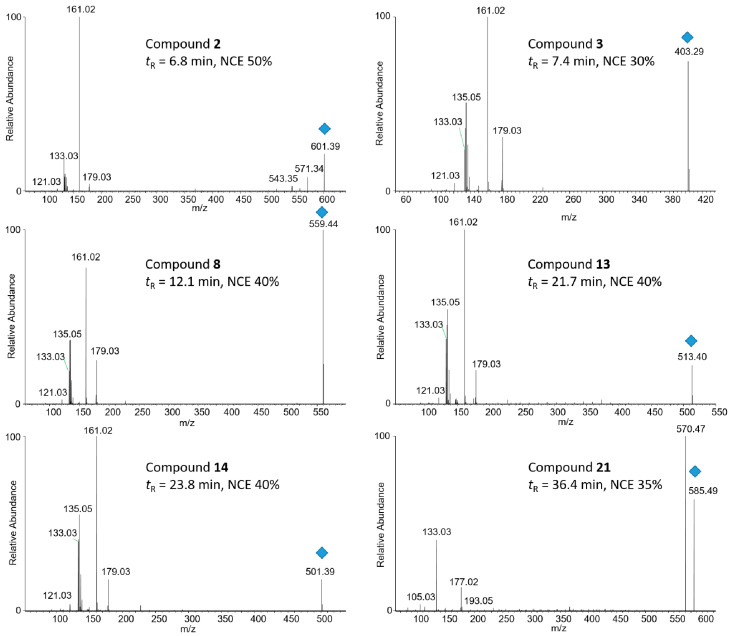
HPLC–ESI^−^-HR-MS/MS spectra of antioxidant *Robinia pseudoacacia* stem bark compounds **2**, **3**, **8**, **13**, **14**, and **21** using normalized collision energies (NCE) ranging from 30 to 50%. Precursor ions were marked with blue rhombuses.

**Figure 6:**
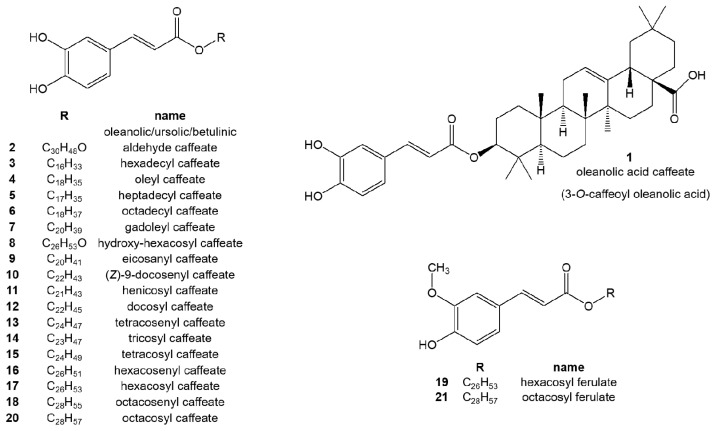
Chemical structures of the identified antioxidant *Robinia pseudoacacia* stem bark compounds. Compounds **2**, **3**, **5**, **8**, **11**, **13**, **14**, **16**, **18**–**21** were identified tentatively.

The 21 antioxidant compounds were further characterized and identified by HPLC–HR-ESI^−^-MS/MS adopting the separation method developed for DPPH–HPLC–DAD–ESI^−^-QMS analysis. The HR-ESI^−^-MS/MS data are presented in Table 1, and representative spectra and the chemical structures of the identified or tentatively identified compounds are depicted in Figure 5 and Figure 6. HR-ESI^−^-MS analysis of the 21 compounds in negative ionization mode yielded mass spectra with intense signals for the corresponding deprotonated molecules [M−H]^−^, allowing the determination of their molecular formulae. These assignments were supported by the high mass accuracy of the Orbitrap analyzer with lock mass correction, with absolute mass errors consistently below 2 ppm for all identified compounds (Table 1). Selecting the deprotonated molecules ([M−H]^−^) as precursor ions, compounds **1**–**18** and **20** displayed highly similar MS/MS fragmentation patterns with the characteristic product ions at *m*/*z* 179.03, *m*/*z* 161.02, *m*/*z* 135.05, *m*/*z* 133.03, and *m*/*z* 121.03 (Figure 5), suggesting the presence of a caffeate moiety in these molecules [24,25]. Caffeic acid (C_9_H_8_O_4_) is detectable by ESI^−^-MS at *m*/*z* 179 corresponding to its deprotonated molecule that undergoes fragmentation via the neutral loss of H_2_O (−18 Da, *m*/*z* 161), CO_2_ (−44 Da, *m*/*z* 135), and HCOOH (−46 Da, *m*/*z* 133) [26], while the daughter ion at *m*/*z* 121 (C_7_H_5_O_2_^–^) is also a diagnostic ion for the caffeic acid [26]. Compounds **1** and **2** showed additional characteristic fragment ions consistent with the sequential losses of two methyl groups (−30 Da) and CO_2_ (−44 Da) or CO (−28 Da), respectively. Compound **1**, namely 3-*O*-caffeoyl oleanolic acid (Figure 6), has already been identified as an antioxidant constituent of black locust bark [22,27]. Its product ion at *m*/*z* 437 corresponds to the deprotonated oleanolic acid after a loss of water ([M−H−H_2_O]^−^). Based on its fragmentation pattern, compound **2** was tentatively identified as one of the structural isomers of 3-*O*-caffeoyl oleanolic, ursolic, or betulinic aldehyde. While 3-*O*-caffeoyl ursolic aldehyde has not yet been described as a natural product, 3-*O*-caffeoyl oleanolic aldehyde has been found in *Celastrus stephanotifolius* (Makino) Makino (hairy oriental bittersweet) dried stems [28] and *Leptopus clarkei* (Hook.fil.) Pojark. [29]. Oleanolic aldehyde caffeate displayed moderate antiproliferative activity against several cancer cell lines, including HepG2, MCF-7, A549, and HeLa, with the phenylpropanoid moiety suggested to play a key role in this effect [29]. The presence of 3-*O*-caffeoyl betulinic aldehyde in dried stems of *C. stehanotifolius* [28] and *Celastrus orbiculatus* Thunb. (Chinese bittersweet) [30], and outer bark of *Betula platyphylla* Sukaczev (white birch) [31] has been published.

By comparing HRMS and HRMS/MS data with those of caffeate esters of fatty alcohols previously isolated and structurally elucidated from methanol extract of black locust stem bark [22], compounds **4**, **6**, **7**, **9**, **10**, **12**, **15**, and **17** were identified with level C confidence according to Çiçek et al. [32] as oleyl caffeate, octadecyl caffeate, gadoleyl caffeate, eicosanyl caffeate, (*Z*)-9-docosenyl caffeate, docosyl caffeate, tetracosyl caffeate, and hexacosyl caffeate, respectively (Table 1, Figure 6). The free radical scavenging activity of these compounds has been previously assessed using a microplate DPPH assay, which showed that compound **1** displayed a stronger antioxidant effect than caffeic acid, with 1.20 mol caffeic acid equivalent/mol. The antioxidant capacities of compounds **4**, **6**, **7**, **9**, **10**, **12**, and **15** ranged from 0.29 to 0.67 mol caffeic acid equivalents per mol of compound, without any structural relationship, i.e., the radical scavenging activity of the caffeate esters did not correlate with either the carbon chain length or their alkyl or alkenyl nature [22]. Similarly, the antioxidant activity of synthetic C_1_–C_16_ alkyl caffeates was similar to that of caffeic acid and independent of the alkyl chain length [33].

The previously obtained results allowed the high-confidence assignments of compounds **1**, **4**, **6**, **7**, **9**, **10**, **12**, **15**, and **17**, while the antioxidant compounds **2**, **3**, **5**, **8**, **11**, **13**, **14**, **16**, **18**–**21** could only be tentatively identified with level E confidence according to Çiçek et al. [32], due to the lack of reference standards and further structural characterization, e.g., by NMR spectroscopy after isolation. Note that the isolation of minor components not appropriately separable from the major components is a significant challenge and was beyond the scope of the present study.

Compounds **3**, **5**, **11**, **14**, and **20** were tentatively identified as the caffeate esters of fatty alcohols, corresponding to hexadecyl, heptadecyl, henicosyl, tricosyl, and octacosyl alcohols, respectively (Table 1, Figure 6), which were reported for the first time in the black locust plant species. However, compound **3** has been described as a component of, e.g., sweet potato (*Ipomoea batatas* (L.) Lam. cv. Beauregard) root [34,35] or acacia (*Acacia dealbata* Link and *A. melanoxylon* R. Br.) bark [36]. Compound **3** exhibited antioxidant effects in both DPPH and ABTS assays [33,37], and inhibited cyclooxygenase-2 (COX-2) enzyme and Fe^2+^-induced lipid peroxidation using large unilamellar vesicles [38]. In addition, its antiproliferative activity has been demonstrated against MCF-7 (breast), SF-268 (central nervous system, CNS), NCI-H460 (lung), HCT-116 (colon), and AGS (gastric) human tumor cells [38,39]. Sweet potato (*I. batatas*) [35] and salt cress (*Eutrema salsugineum* (Pall.) Al-Shehbaz & Warwick ecotype Yukon) [34] roots and aerial parts of trailing ice plant (*Lampranthus spectabilis* N.E.Br.) [40] were found as the source of compound **5**, which did not display significant radical scavenging activity in the DPPH assay [40]. The presence of compounds **11** and **14** was reported, e.g., in the rapeseed (*Brassica napus* L. var. napus cv. Westar) root [34] and Texas mountain laurel (*Dermatophyllum secundiflorum* (Ortega) Gandhi & Reveal, formerly known as *Sophora secundiflora*) stem [41], although no bioactivities have been attributed to them. Compound **20** has been detected in acacia bark [36], aerial parts of soft sophora (*Sophora mollis* (Royle) Graham ex Baker) [42], and root bark of African blackwood (*Dalbergia melanoxylon* Guill. & Perr.) [43], which exhibited mild anti-inflammatory activity by reducing paw edema, a weak anticancer effect against HeLa and 3T3 cell lines, and a strong antioxidant effect in the DPPH radical scavenging assay [42].

The tentatively identified hydroxy-hexacosyl caffeate (**8**), tetracosenyl caffeate (**13**), hexacosenyl caffeate (**16**), and octacosenyl caffeate (**18**) (Figure 6) were identified as new natural products, as verified by database searches in CAS SciFinder^®^ and Reaxys. Compounds **13**, **16**, and **18** are caffeate esters of monounsaturated fatty alcohols, while compound **8** is a caffeate ester of a hydroxylated saturated fatty alcohol. Similarly, in our previous study [22], three caffeate esters of monounsaturated fatty alcohols were tentatively identified as new natural products corresponding to compounds **4**, **7**, and **10** in this study. Furthermore, no literature data on the bioactivity of compounds **8**, **11**, **13**, **14**, **16**, and **18** were found, indicating that this is the first report describing their antioxidant effect.

The MS fragmentation of the deprotonated molecules of compounds **19** (*m*/*z* 557.4573) and **21** (*m*/*z* 585.4895) gave a similar pattern, including the demethylated fragments (at *m*/*z* 542 and *m*/*z* 570, respectively) and product ions at *m*/*z* 193 (C_10_H_9_O_4_^−^, deprotonated molecule of ferulic acid, with very low intensity), *m*/*z* 177 (C_9_H_5_O_4_^−^), *m*/*z* 133 (C_8_H_5_O_2_^−^), and *m*/*z* 105 (C_7_H_5_O^−^) (Table 1, Figure 5), which is typical for ferulate esters in negative ionization mode [44,45]. Thus, compounds **19** and **21** were tentatively identified as hexacosyl ferulate and octacosyl ferulate (Figure 6), respectively, and reported here for the first time in *R. pseudoacacia*. However, among others, croton (*Croton megalocarpus* Hutch) [46] and acacia (*A. dealbata* and *A. melanoxylon*) [36] barks, rice (*Oryza sativa* L.) [34], and cattail (*Typha domingensis* P., and *Typha latifolia* L.) [47] contain compounds **19** and **21** that have displayed antioxidant activity in the DPPH assay, and thrombolytic and cytotoxic effects in brine shrimp lethality bioassay [48]. However, the strength of the antioxidant activity of compound **19** remains uncertain as it has been reported to range from weak to significant in different studies [49,50]. Interestingly, a mixture of ferulate esters of long-chain alcohols (eicosyl, henicosyl, docosyl, tetracosyl, and hexacosyl alcohols) exhibited post-ingestive effects on *Spodoptera littoralis* larvae, acting as strong toxicants, as indicated by the reduction in larval weight and decreased consumption [51]. This mixture also showed moderate cytotoxic activity against Sf9 insect cells, but remained inactive against mammalian Chinese hamster ovary (CHO) cells. Additionally, it strongly inhibited seed germination in lettuce (*Lactuca sativa* L.) [51].

## 3. Materials and Methods

### 3.1. Chemicals

All chemicals were used as received from commercial suppliers without further purification. Solvents of analytical grade (methanol, ethanol, ethyl acetate, *n*-hexane) were obtained from Molar Chemicals (Halásztelek, Hungary). For HPLC–MS analyses, acetonitrile (MS grade) and formic acid (MS grade) were purchased from VWR (Debrecen, Hungary), while ethanol (HPLC grade) from Molar Chemicals. Ultrapure water was prepared by the Millipore Direct-Q 3 UV Water Purification System (Merck, Darmstadt, Germany). The 2,2-diphenyl-1-picrylhydrazyl radical (DPPH) was acquired from Sigma-Aldrich (Steinheim, Germany).

### 3.2. Sample Origin and Preparation

*Robinia pseudoacacia* stem bark was collected from a flowering tree in May 2026 in Harta (46°41′45″ N 19°02′26″ E, altitude: 93 m a. s. l.) in the Great Plain of Hungary. The plant material was identified by Ágnes M. Móricz, and a voucher specimen (PPI-MA-RB2) has been deposited at the herbarium of the Plant Protection Institute, Centre for Agricultural Research, Budapest, Hungary. The sample was air-dried at room temperature, protected from direct sunlight, and powdered using a coffee grinder (Sencor SCG 2050, Říčany, Czech Republic). Three separate 500 mg portions of each dried, ground sample were independently extracted with 5 mL of methanol, ethyl acetate, or *n*-hexane in 20 mL screw-capped vials using ultrasound-assisted extraction for 10 min at room temperature (Sonorex Super RK 106, Bandelin, Berlin, Germany). After sedimentation for 5 min, the supernatant (4 mL) of each extract was filtered through a 0.22 µm polytetrafluoroethylene (PTFE) syringe filter (FilterBio, Nantong, China) and evaporated to dryness using a sample concentrator at 40 °C under a gentle stream of nitrogen (MD200-2, Hangzhou Allsheng Instruments, Hangzhou, China). The dried residues were reconstituted in 1 mL of ethanol, filtered again through a 0.22 µm PTFE syringe filter (FilterBio), and immediately analyzed.

### 3.3. DPPH-HPLC–DAD–ESI-QMS

The ethyl acetate and methanol extracts were diluted ten-fold, while the *n*-hexane extract was diluted two-fold. The dilutions were performed using ethanol (as a control) or an ethanol solution of DPPH to reach a final DPPH concentration of 0.1 mg/mL. The mixtures were incubated for 10 min at room temperature in the dark prior to analysis. The screening for antioxidant components of the extracts was carried out using an HPLC–DAD–ESI-QMS system (LCMS-2020, Shimadzu, Kyoto, Japan). Separation was performed before and after DPPH treatment at 35 °C on a Reprospher 100 C18-DE column (150 mm × 3 mm ID, 5 μm particle size; Dr. Maisch, Ammerbuch, Germany) using a mobile phase flow rate of 0.5 mL/min and an injection volume of 5 μL. The gradient of 5% aqueous acetonitrile containing 0.1% (*V*/*V*) formic acid (solvent A) and ethanol containing 0.1% (*V*/*V*) formic acid (solvent B) was as follows: 0–10 min, 83% B; 10–30 min, 83–92% B; 30–39 min, 92–100% B; 39–42 min, 100% B; 42–45 min, 83% B. The DAD acquired UV-Vis spectra in the range of 190–800 nm with a slit width of 1.2 nm, and HPLC–UV chromatograms were monitored at 325 nm for phenolic compounds. MS detection was conducted under the following conditions: desolvation line (DL) temperature, 250 °C; heat block temperature, 400 °C; interface temperature, 350 °C; drying nitrogen gas flow, 15 L/min; nebulizer nitrogen gas flow, 1.5 L/min; negative ionization mode. The full-scan ESI^−^–QMS spectra were acquired in the range of *m*/*z* 150–900 with a scan speed of 5000 u/s. Instrument control and data acquisition were performed using LabSolutions 5.72 software (Shimadzu). Antioxidant activity was evaluated by comparing the peak areas obtained by integrating the HPLC–ESI-QMS extracted ion chromatograms (EICs) of the compounds from the untreated (control) and DPPH-treated samples. A reduction in peak area indicated radical scavenging activity.

### 3.4. HPLC–HR-ESI-MS/MS

The same chromatographic conditions described in Section 3.3 were used for HPLC–HR-ESI-MS/MS analyses using the combination of a UHPLC system (Vanquish Flex VF-P10, Dionex Softron, Germering, Germany) and a hybrid quadrupole-Orbitrap mass spectrometer with a HESI-II probe (Orbitrap Exploris 120, Thermo Fisher Scientific, Bremen, Germany). The injection volume was 1 µL. The ESI source was operated in negative ionization mode, and the working parameters were as follows: spray voltage, −2.0 kV; capillary temperature, 320 °C; vaporizer temperature, 250 °C; sheath and auxiliary gas (N_2_, produced by a Peak Scientific gas generator, Genius XE 35, Glasgow, UK), 10 and 5 arbitrary units, respectively. Full-scan HRMS spectra were recorded with a resolution of 120,000 in the scan range of *m*/*z* 400–620 using EASY-IC (fluoranthene radical ions) lock mass correction in scan-to-scan mode. The top two most intense ions detected in either full-scan or selected ion monitoring (SIM) mode were selected for data-dependent MS/MS (ddMS^2^) scan using a quadrupole isolation window of *m*/*z* 0.5 and a resolving power of 30,000 in the auto scan range mode, without lock mass correction. Precursor ions were fragmented in higher-energy collisional dissociation (HCD) fragmentation mode with normalized collision energies (NCE) of 30%, 35%, 40%, and 50%. Automatic gain control (AGC) target and maximum injection times (IT) were set to standard and auto, respectively, for both full-scan and ddMS^2^ experiments. Instrument control, operation, and data processing were performed with Xcalibur 4.7.69 software (Thermo Fisher Scientific).

## 4. Conclusions

Antioxidant screening using an off-line DPPH assay combined with HPLC (DPPH–HPLC) revealed 21 amphipathic phenolic compounds with radical scavenging activity from the stem bark extracts of black locust (*Robinia pseudoacacia*). HPLC–HR-ESI^−^-MS/MS analyses of the 21 compounds resulted in the identification or tentative identification of two caffeate esters of triterpenes, 12 caffeate esters of saturated fatty alcohols, six caffeate esters of monounsaturated fatty alcohols, one caffeate ester of a saturated hydroxylated fatty alcohol, and two ferulate esters of saturated fatty alcohols. Among them, four compounds—hydroxy-hexacosyl caffeate (**8**), tetracosenyl caffeate (**13**), hexacosenyl caffeate (**16**), and octacosenyl caffeate (**18**)—were tentatively identified as previously undescribed natural products. Eight additional compounds, namely 3-*O*-caffeoyl oleanolic, ursolic, or betulinic aldehyde (**2**), and hexadecyl (**3**), heptadecyl (**5**), henicosyl (**11**), tricosyl (**14**), and octacosyl (**20**) caffeates, and hexacosyl (**19**) and octacosyl (**21**) ferulates, were reported here for the first time as the components of *R. pseudoacacia* plant. Moreover, the antioxidant activity was attributed to the novel compounds **8**, **13**, **16**, and **18**, as well as to the known compounds **11** and **14** for the first time. These results indicate that black locust stem bark represents a promising, rich source of antioxidant specialized metabolites. The presence of these long-chain caffeate and ferulate esters provides a basis for future studies investigating their functional properties in lipid-rich environments. Future research should address structure–activity relationships and antioxidant performance in biologically relevant systems to better evaluate their mechanisms of action before any practical applicability can be assessed.

## Figures and Tables

**Table 1:** HPLC–HR-ESI^−^-MS/MS data of the antioxidant compounds (**1**–**21**) detected in *Robinia pseudoacacia* stem bark extracts.

Compounds	*t*_R_ (min)	Precursor Ion [M−H]^−^ (*m*/*z*) Experimental	Error (ppm)	Molecular Formula	Normalized Collision Energy (%)	Fragment Ions (*m*/*z*)	Names
**1**	3.5	617.3846	−0.26	C_39_H_54_O_6_	50	587, 543, 437, 179, 161, 135, 133, 121	3-*O*-caffeoyl oleanolic acid
**2**	6.8	601.3898	−0.08	C_39_H_54_O_5_	50	571, 543, 179, 161, 135, 133, 121	3-*O*-caffeoyl oleanolic/betulinic/ursolic aldehyde *
**3**	7.4	403.2847	−1.70	C_25_H_40_O_4_	30	179, 161, 135, 133, 121	hexadecyl caffeate *
**4**	7.9	429.3006	−1.01	C_27_H_42_O_4_	30	179, 161, 135, 133, 121	oleyl caffeate
**5**	9.5	417.3003	−1.76	C_26_H_42_O_4_	30	179, 161, 135, 133, 121	heptadecyl caffeate*
**6**	10.9	431.3159	−1.82	C_27_H_44_O_4_	30	179, 161, 135, 133, 121	octadecyl caffeate
**7**	11.5	457.3319	−0.95	C_29_H_46_O_4_	30	179, 161, 135, 133, 121	gadoleyl caffeate
**8**	12.1	559.4364	−0.71	C_35_H_60_O_5_	40	179, 161, 135, 133, 121	hydroxy-hexacosyl caffeate *
**9**	15.9	459.3472	−1.71	C_29_H_48_O_4_	40	179, 161, 135, 133, 121	eicosanyl caffeate
**10**	16.6	485.3634	−0.48	C_31_H_50_O_4_	40	179, 161, 135, 133, 121	(*Z)*-9-docosenyl caffeate
**11**	18.4	473.3632	−0.92	C_30_H_50_O_4_	40	179, 161, 135, 133, 121	henicosyl caffeate *
**12**	21.1	487.3788	−0.99	C_31_H_52_O_4_	40	179, 161, 135, 133, 121	docosyl caffeate
**13**	21.7	513.3947	−0.46	C_33_H_54_O_4_	40	179, 161, 135, 133, 121	tetracosenyl caffeate *
**14**	23.8	501.3944	−1.06	C_32_H_54_O_4_	40	179, 161, 135, 133, 121	tricosyl caffeate *
**15**	26.1	515.4103	−0.55	C_33_H_56_O_4_	40	179, 161, 135, 133, 121	tetracosyl caffeate
**16**	26.7	541.4261	−0.25	C_35_H_58_O_4_	40	179, 161, 135, 133, 121	hexacosenyl caffeate *
**17**	30.6	543.4414	−0.89	C_35_H_60_O_4_	40	179, 161, 135, 133, 121	hexacosanyl caffeate
**18**	31.1	569.4572	−0.59	C_37_H_62_O_4_	40	179, 161, 135, 133, 121	octacosenyl caffeate *
**19**	33.5	557.4573	−0.42	C_36_H_62_O_4_	35	542, 193, 177, 133, 105	hexacosyl ferulate *
**20**	34.4	571.4730	−0.32	C_37_H_64_O_4_	40	179, 161, 135, 133, 121	octacosyl caffeate *
**21**	36.4	585.4895	1.14	C_38_H_66_O_4_	35	570, 193, 177, 133, 105	octacosyl ferulate *

* tentative identification.

## Data Availability

The data supporting the findings of this study are available from the corresponding author upon reasonable request.
